# Hematological Effects and Benchmark Doses of Long-Term Co-Exposure to Benzene, Toluene, and Xylenes in a Follow-Up Study on Petrochemical Workers

**DOI:** 10.3390/toxics10090502

**Published:** 2022-08-28

**Authors:** Zhaorui Zhang, Xin Liu, Chaofan Guo, Xinjie Zhang, Yingying Zhang, Na Deng, Guanchao Lai, Aichu Yang, Yongshun Huang, Shanfeng Dang, Yanqun Zhu, Xiumei Xing, Yongmei Xiao, Qifei Deng

**Affiliations:** 1 Department of Occupational and Environmental Health, School of Public Health, Sun Yat-sen University, Guangzhou 510080, China; 2 Guangdong Provincial Key Laboratory of Occupational Disease Prevention and Treatment, Guangdong Province Hospital for Occupational Disease Prevention and Treatment, Guangzhou 510300, China; 3 Occupational Disease Prevention and Treatment Institute of Sinopec Maoming Petrochemical Company, Maoming 525000, China; 4 School of Public Health, Guangzhou Medical University, Guangzhou 511436, China

**Keywords:** BTX components, occupational co-exposure, decline in hematologic parameters, BMD estimation

## Abstract

Benzene, toluene, and xylenes (BTX) commonly co-exist. Exposure to individual components and BTX-rich mixtures can induce hematological effects. However, the hematological effects of long-term exposure to BTX are still unclear, and respective reference levels based on empirical evidence should be developed. We conducted a follow-up study in BTX-exposed petrochemical workers. Long-term exposure levels were quantified by measuring cumulative exposure (CE). Generalized weighted quantile sum (WQS) regression models and Benchmark Dose (BMD) Software were used to evaluate their combined effects and calculate their BMDs, respectively. Many hematologic parameters were significantly decreased at the four-year follow-up (*p* < 0.05). We found positive associations of CE levels of benzene, toluene, and xylene with the decline in monocyte counts, lymphocyte counts, and hematocrit, respectively (β > 0.010, *P*_trend_ < 0.05). These associations were stronger in subjects with higher baseline parameters, males, drinkers, or overweight subjects (*P*_interaction_ < 0.05). BTX had positive combined effects on the decline in monocyte counts, red-blood-cell counts, and hemoglobin concentrations (*P*_trend_ for WQS indices < 0.05). The estimated BMDs for CE levels of benzene, toluene, and xylene were 2.138, 1.449, and 2.937 mg/m^3^ × year, respectively. Our study demonstrated the hematological effects of long-term BTX co-exposure and developed 8h-RELs of about 0.01 ppm based on their hematological effects.

## 1. Introduction

Benzene, toluene, and xylenes (BTX) are representative single-ring aromatic compounds. They are ubiquitous coexistent in human environments due to their high volatility and extensive emission sources. The major sources are vehicle exhaust, cigarette smoke, and the emission of BTX compounds and their related products, such as petrochemicals, pharmaceutical products, and a variety of solvents used in adhesives, paints, and rubber products [1]. The recent accelerating development of industrialization in many developing countries, including China, has led to the increase in the production of BTX-related petrochemical products and long-term exposure of petrochemical workers to relatively higher BTX levels.

Highly volatile BTX can be readily absorbed after inhalation, extensively metabolized by cytochrome P450 enzymes (CYPs), and distributed to lipid-rich and highly vascular tissues (such as bone marrow) due to their lipophilicity [2]. In addition to their common physicochemical characteristics and metabolic processes, BTX can also cause some similar adverse health effects with chronic exposure [2]. For instance, chronic benzene exposure induces the arrested development of blood cells, aplastic anemia, and leukemia [2,3]. The International Agency for Research on Cancer has classified benzene as a Group 1 carcinogen [2]. The effects of toluene and xylenes on hematologic parameters have recently drawn more and more attention [4,5,6,7]. However, the epidemiological evidence of the hematological effects induced by individual BTX components is not quite conclusive, probably because of the confounding effects of other co-existing BTX components [8].

Multiple pollutants in human environments may cause adverse effects in an additive, synergistic, or antagonistic manner [8,9]. Increasing evidence suggests that the toxic behaviors of individual BTX components can be modified by other co-existing BTX components due to their competitive metabolic interactions [8]. For example, benzene-induced adverse effects can be modified by toluene [8,10]. Previous epidemiologic studies have shown hematological responses to BTX-rich mixtures [5,6]. However, the combined hematological effects of BTX components remain unclear.

To reduce BTX-related adverse effects, many countries have established or recommended various environmental and occupational exposure limits and taken effective actions to reduce BTX (especially benzene) concentrations in the air. However, significant hematological effects have still been found in individuals chronically exposed to benzene at concentrations lower than 1 ppm, a commonly recommended occupational exposure limit [11,12,13]. Moreover, the existing mandatory or recommended exposure limits for toluene and xylene are established mainly based on their non-hematological adverse effects. For instance, the U.S. Environmental Protection Agency (EPA) has established a Reference Concentration (RfC) of 5 mg/m^3^ for toluene, at which adverse neurological effects would not be expected from a lifetime inhalation exposure in humans [14]. The Agency for Toxic Substances and Disease Registry (ATSDR) has developed a chronic inhalation minimal risk level (MRL) of 0.4 mg/m³ for xylene based on its neurological effects in occupationally exposed workers [15]. Thus, it is important to propose a more sensitive reference exposure level (REL) for benzene and to fill the research gap of RELs for toluene and xylene based on their hematological effects, especially in occupationally exposed workers who probably have an increased risk of hematological effects. Previously, RELs were determined considering the point of departure (POD) using no-observed-adverse-effect levels (NOAELs) or lowest-observed-adverse-effect levels (LOAELs). Recently, the benchmark dose (BMD) approach is highly recommended as the preferred method for calculating RELs, since it incorporates and conveys more information than NOAELs or LOAELs in determining the POD [16]. The BMD is the dose estimated using fitted dose–response curves that produces a predetermined benchmark response (BMR), such as a 10% increase in the incidence of a particular health effect. The BMD’s lower confidence limit (BMDL) is used as the POD in determining RELs. 

Occupational longitudinal studies may provide a deeper understanding of hematological effects associated with long-term BTX co-exposure. Therefore, we conducted a follow-up study in petrochemical workers who had been long-termly co-exposed to BTX components. Their occupational BTX exposure levels were quantified using cumulative exposure (CE). The decline in hematologic parameters was defined as the baseline parameters minus the corresponding values in the follow-up stage. We evaluated the single and combined effects of BTX components on the decline in hematologic parameters. We set criteria to define hematological damage as the health endpoint for BMD calculation. Finally, we estimated the BMD and the BMDL for individual BTX components and proposed 8 h-RELs that would not increase the risk of hematological damage for repeated daily 8 h exposure over a significant fraction of a lifetime [17].

## 2. Materials and Methods

### 2.1. Study Population

The study participants were recruited in 2011–2012 from two large-scale state-owned petrochemical companies in Guangzhou and Maoming, Guangdong Province, China. Considering that the co-existence of other occupational hazards, such as noise and other chemical toxicants, may also induce health effects, we consulted occupational health specialists and systematically evaluated the annual hazard information in the workplaces for three years before initiating the study to identify the primary hazards. BTX were the primary occupational hazards in 32 workplaces of two petrochemical companies, including petroleum refining, chemical production, and petroleum processing. 

At baseline, participants from these 32 workplaces were selected based on the following criteria: (a) at least one year of employment; (b) no previous self-reported diagnosis of cancers, cardiopulmonary diseases, hematological diseases, chronic immune diseases, and/or diabetes; (c) not taking medicine or receiving X-ray tests one week before the baseline investigation; (d) no obvious abnormities in hematologic parameters in baseline physical examination; and e) providing completed occupational questionnaire, physical examinations, and sufficient biological samples. A total of 1443 participants were enrolled at baseline. Among them, 1054 subjects (follow-up rate: 73.04%) completed the follow-up survey in 2015–2016. Of the remaining 389 subjects that were not successfully followed up, 3 had developed serious diseases; 5 had taken medicine and/or X-ray examination one week before the follow-up investigation; 11 were unwilling or unable to complete any parts of the survey for personal or business reasons; the other 370 did not provide sufficient or eligible EDTA-anticoagulated blood samples. This follow-up study was approved by the Ethical Review Committee of School of Public Health, Sun Yat-sen University (ethical approval code: [2011]-35).

In both stages, each participant signed a written informed consent form and then was interviewed face-to-face by trained personnel. A pretested occupational questionnaire was used to collect general information, including demographic characteristics, lifestyle (such as smoking and drinking status), medical and medication history, and occupational experience (such as workplaces and work years). Smokers were defined as smoking ≥1 cigarette per day for ≥1 year in their lifetime, and drinkers were defined as having drunk alcoholic beverages more than once per week for ≥1 year in their lifetime. After the interview, EDTA-anticoagulated fasting venous blood was collected for hematologic parameter detection.

### 2.2. Assessment of Individual BTX Exposure Levels

Ambient BTX concentrations in the workplaces of participants were seasonally monitored for three years before the baseline stage. Sampling in each workplace was performed according to “Sampling Specification for Monitoring of Hazardous Substances in the Air of the Workplace” (GBZ 159-2004) (Table S1). Air samplers (Sp1500; TSI Corporation Shoreview, MN, USA) and active-carbon tubes were used to collect duplicate air samples at a flow rate of 50 mL/min for 2~8 h in the personal breathing zone. Thermal desorption and capillary gas chromatography coupled with a hydrogen flame ionization detector (Agilent 6890; Palo Alto, CA, USA) were then used to determine BTX concentrations according to National Institute for Occupational Safety and Health (NIOSH) method 1501. The limits of detection (LODs) for the ambient concentrations of benzene, toluene, and xylene were 0.01 mg/m^3^, 0.02 mg/m^3^, and 0.02 mg/m^3^, respectively, and the values below the LODs were replaced by LOD/2 (Table S1). Eight-hour time-weighted average (8 h-TWA) concentrations were calculated. To evaluate long-term occupational BTX exposure levels for all participants, we calculated the CE levels (mg/m^3^ × year) of BTX components by multiplying their work years by the three-year mean 8h-TWA concentrations in workplaces.

### 2.3. Detection of Hematologic Parameters

Nine hematologic parameters were measured in both stages, including white blood cell (WBC) counts, neutrophil counts, monocyte counts, lymphocyte counts, red blood cell (RBC) counts, hemoglobin concentrations, hematocrit, platelet counts, and mean platelet volume (MPV). Blood samples were sent to a professional clinical laboratory within two hours of collection for hematologic parameter detection using an automatic hematology analyzer (Sysmex XE-2100; Chuo-ku Kobe, Hyogo, Japan). Each blood sample was assayed in duplicate, and the average values were used for statistical analyses. The decline in hematologic parameters was defined as the baseline levels minus the corresponding levels measured in the follow-up stage.

### 2.4. Definition of Hematological Damage

As there are no universally recognized definitions for hematological damage exclusive to BTX exposure, we proposed some criteria to facilitate BMD calculation. To increase the sensitivity, we defined hematological damage based on not only the levels of the parameters detected in the follow-up stage but also the four-year decline in hematologic parameters. Furthermore, to increase the specificity and reduce the false-positive rate, subjects with at least two parameters that were abnormally low in the follow-up stage or/and with at least two parameters that had an abnormally high four-year decline were classified as cases of hematological damage. Thus, hematological damage had to meet at least one of the following criteria: (1) at least two hematologic parameters (except for MPV, which has no related national standards) in the follow-up stage were below the lower limits of the corresponding normal range in Chinese adults according to “Reference Intervals for Blood Cell Analysis” (WS/T 405-2012) (Table S2); (2) at least two hematologic parameters with the magnitude of decline higher than the 95th percentile of the population of the same gender.

### 2.5. Statistical Analysis

BTX CE levels were natural logarithm (ln) transformed to be a normal distribution. The covariates at baseline included age (continuous), sex (male/female), factory location (Guangzhou/Maoming), body mass index (BMI; continuous), smoking status (smokers/non-smokers), pack-years of smoking (continuous), drinking status (drinkers/non-drinkers), and the corresponding baseline parameters (continuous). We included these covariates in statistical analyses unless otherwise specified.

The differences in hematologic parameters between baseline and follow-up stages were compared using paired Student’s *t*-tests. We used a multivariable covariance analysis with adjustment for other covariates to compare the magnitude of decline between subjects with different general characteristics, including age (≤40 vs. >40, stratified by median age), sex (female vs. male), smoking status (non-smokers vs. smokers), drinking status (non-drinkers vs. drinkers), and BMI (<24 kg/m^2^ vs. ≥24 kg/m^2^, stratified by overweight standard commonly used in Chinese adults). The associations of individual BTX components with the decline in hematologic parameters were analyzed by covariate-adjusted linear regression models. To provide more information for risk assessment, we performed an analysis stratified by baseline hematologic parameters. The total population was divided into three groups (low, medium, and high) according to the tertiles of baseline hematologic parameters. We also performed an analysis stratified by general characteristics, including age, sex, smoking status, drinking status, and BMI. The effect modification of baseline parameters and general characteristics on the associations between BTX and the decline in hematologic parameters was evaluated by additionally including an interaction term of each BTX component (continuous) and each stratified variable (categorical) in covariate-adjusted linear regression models. 

Generalized weighted quantile sum (WQS) regression was used to evaluate BTX combined effects and to identify the contribution of each component. WQS regression is a dimension-reduction method that transforms multiple correlated variables into one independent index (i.e., the WQS index). Then, the generated WQS index is included in regression models to assess the combined effects of multiple variables [18]. In this study, we scored each BTX component into quartiles and split the data into training and validation sets as 40%/60% to test the statistical significance of the WQS indices and estimate the WQS index weights (*w*) for each component. We conducted a *B* = 100 bootstrap sampling to estimate the stability selection of *w* by maximizing the likelihood for models. We estimated the combined effects of BTX components according to the covariate-adjusted associations between their WQS indices and the decline in hematologic parameters and identified the contribution of each component based on its weight (*w*) in the WQS indices.

To estimate the BMD, we divided the subjects into 7–8 groups according to the CE distributions of the BTX components to ensure that each group had a clear CE range and an appropriate sample size ranging from 70 to 200 (Table S3). The exposure level in each group was the median CE value of the BTX components (Table S3). The BMDs and BMDLs were estimated using Benchmark Dose Software (BMDS 3.20; EPA, USA) according to the technical guidance of the U.S. EPA (EPA/100/R-12/001) [16]. The BMR was set at a default value of 10% extra risk of hematological damage. We tested all models for dichotomous data, including Dichotomous Hill, Gamma, Log-Logistic, Multistage, Weibull, Logistic, Log Probit, and Probit. We selected the models that adequately fit our data (i.e., *p* for goodness-of-fit tests > 0.1) and then picked the best model according to the following criteria recommended by EPA guidance: a BMD not higher than the maximum dose; BMD/BMDL <3; and the lowest Akaike’s Information Criterion (AIC).

Generalized WQS regression and BMD estimation were performed using the gWQS package in R (version 4.0.5) and BMDS (Version 3.20), respectively, and other analyses were conducted using SPSS statistical software package 20.0 (Chicago, IL, USA). All statistical tests were two-sided, and *p* < 0.05 was considered to be statistically significant.

## 3. Results

### 3.1. Ambient BTX 8h-TWA Concentrations and Subject Characteristics

A total of 510 duplicate ambient samples of the workplaces were collected (Table S1). The median (25th percentile, 75th percentile) 8h-TWA concentrations of benzene, toluene, and xylene were 0.012 (0.012, 0.032) mg/m^3^, 0.024 (0.024, 0.048) mg/m^3^, and 0.068 (0.068, 0.094) mg/m^3^, respectively, and were significantly modestly correlated with each other (Spearman correlation coefficients: 0.440–0.502; *p* < 0.001) (Table S4). The annual average 8h-TWA concentrations of BTX components were not significantly different (*p* > 0.05) (Figure S1), suggesting that BTX concentrations in the workplaces were relatively stable. Thus, we used the average 8h-TWA concentrations in a three-year period to calculate the BTX CE levels for individual participants.

Table 1 shows the baseline general characteristics, BTX CE levels, and hematologic parameters in both stages. Participants were relatively young (mean age: 39.91) and had a long duration of occupational BTX exposure (mean work years: 18.75). The majority of participants were male (77.42%) and non-smokers (67.46%). The median CE levels of benzene, toluene, and xylene were 0.65 mg/m^3^ × year, 0.84 mg/m^3^ × year, and 1.37 mg/m^3^ × year, respectively. Monocyte counts, lymphocyte counts, RBC counts, hemoglobin concentrations, and MPV were significantly decreased at the the four-year follow-up (*p* < 0.05), with their mean decline values being greater than 0 (Table 1). After adjusting for other primary covariables, some parameters showed a greater decline in males, subjects over the age of 40, smokers, or subjects with a BMI <24 kg/m^2^ (*p* < 0.05) (Table S5). 

### 3.2. Associations of Individual BTX Components with Decline in Hematologic Parameters

After adjustment for multiple covariates, benzene CE levels were positively associated with the decline in monocyte counts (β = 0.012, *P*_trend_ = 0.007); toluene CE levels were positively associated with the decline in lymphocyte counts (β = 0.047, *P*_trend_ = 0.021); and xylene CE levels were positively associated with the decline in hematocrit (β = 0.259, *P*_trend_ = 0.032) (Table 2). 

To provide more information for risk assessment, we further performed analyses stratified by baseline parameters and general characteristics (Table 3 and Tables S6–S11). Interestingly, we observed positive associations of BTX with the decline in hematologic parameters, which were mostly stronger in subjects with higher baseline levels, males, drinkers, or subjects with a BMI ≥ 24 kg/m^2^ (*P*_interaction_ < 0.05) (Table 3). Specifically, the associations of xylene with the decline in monocyte counts (β = 0.053), toluene and xylene with the decline in RBC counts and hematocrit (β > 0.055), and toluene with the decline in hemoglobin concentrations (β = 1.647) were significant and more pronounced in participants with higher baseline values (*P*_interaction_ < 0.05). The associations of xylene with the decline in lymphocyte counts, RBC counts, and hemoglobin concentrations (β > 0.030), and of all BTX components with the decline in hematocrit (β ≥ 0.200) were significant and more pronounced in males (*P*_interaction_ < 0.05). The associations of toluene or/and xylene with the decline in lymphocyte counts (β > 0.065) were significant and more pronounced in non-drinkers or workers with a BMI ≥2 4 kg/m^2^ (*P*_interaction_ < 0.05) (Table 3). 

### 3.3. Combined Effects of BTX on the Decline in Hematologic Parameters

We further evaluated the combined effects of BTX on the decline in hematologic parameters and identified the contribution of each component using WQS regression models (Table 4). We found that BTX had positive combined effects on the decline in monocyte counts, RBC counts, and hemoglobin concentrations (*P*_trend_ for WQS indices < 0.05). Specifically, a quartile increase in the WQS_MOE_ index was associated with a decline of 0.013 × 10^9^/L in monocyte counts (*P*_trend_ = 0.007), and benzene was the most highly weighted component (*w* = 0.976) in the WQS_MOE_ index. A quartile increase in the WQS_RBC_ index was associated with a decline of 0.045 × 10^12^/L in RBC counts (*P*_trend_ = 0.002), and toluene was the most highly weighted component (*w* = 0.614) in the WQS_RBC_ index. In addition, a quartile increase in the WQS_Hb_ index was associated with a decline of 0.972 g/L in hemoglobin concentrations (*P*_trend_ = 0.040), and xylene was the most highly weighted component (*w* = 0.847) in the WQS_Hb_ index.

### 3.4. BMD Estimation

According to the criteria for hematological damage (see Section 2.4), 171 subjects were classified into cases with hematological damage, and the incidence proportions of hematological damage in each BTX exposure group are shown in Table S3. According to EPA guidance (see Section 2.5), the Multistage model, Dichotomous Hill model, and Probit model were considered the best models for benzene, toluene, and xylene, respectively (Table 5). At 10% of the BMR level, the BMDs (BMDLs) for the CE levels of benzene, toluene, and xylene were 2.138 (1.559) mg/m^3^ × year, 1.449 (1.325) mg/m^3^ × year, and 2.937 (2.312) mg/m^3^ × year, respectively (Table 5). The dose–response curves for BTX components are shown in Figure S2. Considering the significant variation in the working duration and the necessity to protect BTX-exposed workers, we calculated the reference 8h-TWAs by dividing the estimated BMDLs by 40 years, which might be a relatively long working duration for workers if employed at 20 years old and retired at 60 years old (i.e., the legal retirement age in China). The reference 8h-TWAs for benzene, toluene, and xylene were 0.0390 mg/m^3^ (about 0.0125 ppm), 0.0331 mg/m^3^ (about 0.0088 ppm), and 0.0578 mg/m^3^ (about 0.0122 ppm), respectively.

## 4. Discussion

In the present follow-up study of BTX-exposed petrochemical workers, we found a significant decrease in many hematologic parameters after a four-year follow-up. Exposure to benzene, toluene, and xylene was positively associated with the decline in monocyte counts, lymphocyte counts, and hematocrit, respectively. The associations between BTX and the decline in hematologic parameters were stronger in subjects with higher baseline parameters, males, drinkers, or overweight. WQS regression models suggested positive combined effects of BTX on the decline in monocyte counts, RBC counts, and hemoglobin concentrations. These findings provide further evidence of the complex hematological effects of long-term occupational BTX co-exposure. We then estimated the BMD and the BMDL for each BTX component to propose 8h-RELs based on their hematological effects.

The most commonly used methods for exposure assessment in occupational populations include external environmental monitoring and internal exposure biomarker detection. BTX components and their metabolites in the biological fluids of exposed subjects have relatively short half-lives [19], which makes it difficult to assess long-term exposure. Although personal sampling is a more precise exposure assessment method, it is not feasible in a follow-up study with a relatively large sample size. Lee et al. [20] reported that there were no differences in the measurement of BTX levels using personal and area sampling methods. Thus, we chose the area sampling method to measure the long-term TWA concentrations of BTX components in the workplaces of our participants. We found that the annual average TWA concentrations of all BTX components were relatively stable. Thus, we calculated BTX CE levels based on the average 8h-TWA concentrations and work years.

We found that many hematologic parameters were significantly reduced at the four-year follow-up, which might have been causes by cumulative exposure to hemotoxins or/and the aging-associated functional alternation in hematopoietic stem cells (HSCs). The petrochemical industry is a major source of multiple hazardous and toxic air pollutants. Mounting evidence has linked the petrochemical industry and residential petrochemical exposure with the increased risk of hematological malignancies, especially leukemia [21,22]. Khuder et al. [23] conducted a small cohort study on 105 petrochemical workers and observed that whole-blood counts, except for WBC counts, significantly decreased during the follow-up period. Our larger follow-up study also observed significant reductions in many hematologic parameters, further highlighting the importance of monitoring hematologic parameters in health surveillance for petrochemical workers. Furthermore, the biological age increased during the follow-up period, which may have caused the deregulation of HSC homeostasis and decline in hematopoiesis, and eventually produced adverse hematological effects such as an increased incidence of anemia and hematological malignancies [24,25]. Our findings might provide further epidemiologic evidence of the aging-related decline in hematopoiesis characterized by decreased hematologic parameters. We also observed significant sex differences in the magnitude of the decline in hematologic parameters. It is widely known that there are biological and behavioral differences between females and males, which affect the incidence, prognosis, and mortality of multiple diseases, including hematological malignancies [26]. 

BTX are important toxic substances in the releases from the petrochemical industry. Thus, we further explored their associations with the decline in hematologic parameters. We observed a positive association of benzene with the decline in monocyte counts. Benzene is the most toxic BTX compound, which has been widely recognized to induce hematotoxicity in humans [27]. One of the important signs of benzene-induced hematotoxicity is the reduction in all types of leukocytes (including monocytes), even at a low dose (<1 ppm) [12,27,28]. Our study also suggested that benzene might be a risk factor for hematopoietic suppression. However, the widely reported relations of benzene with WBC counts were not found in our longitudinal analysis, which may be attributed to the relatively short follow-up period, during which the effects of benzene on WBC counts might have not yet presented. In addition to benzene, the effects of toluene and xylene on hematologic parameters have also been paid more attention to in recent years [4,5,6,7]. In the present study, we found a positive association of toluene exposure with the decline in lymphocyte counts. Tanigawa et al. [29] conducted a panel study on 16 male rotogravure printers and found that exposure to mixed organic solvents (primarily toluene) induced recoverable decreases of T lymphocytes. Win-Shwe et al. [30] also found that the percentage of splenic T lymphocytes was significantly suppressed in male mice exposed to toluene on postnatal days 8–12. We also observed a positive association of xylene exposure with the decline in hematocrit. Chen et al. [5] showed that the blood concentrations of m-/p-xylene were inversely associated with hematocrit among elderly residents living near petrochemical complexes. A study using animal models also found that xylene might reduce hematocrit [31]. Together, these findings suggested that toluene and xylene may have the potential to induce hematotoxicity. However, current evidence regarding their hematological effects is relatively limited [15,32], and further investigations are warranted to look at their hematological effects in more depth.

To provide more information for risk assessment, we evaluated the effect modification of baseline hematological conditions and general characteristics on the hematological effects of BTX components. We interestingly found that the hematological effects of BTX were mostly stronger in subjects with higher baseline levels, indicating that subjects with higher baseline levels of hematologic parameters might be more sensitive to BTX-related hematological effects. We also found that the hematological effects of BTX were mostly stronger in males, implying that males might be more predisposed to BTX-associated hematotoxicity. Previous data showed that males had an increased risk and a poorer prognosis for leukemia than females [26]. These sex differences may be related to different environmental exposure, lifestyles, endogenous hormones, sex chromosomes, epigenetics, or the complex multidirectional interactions among these factors [26]. In addition, Wang et al. [33] found that females had higher levels of urinary metabolites than males for the same environmental exposure to benzene, which may have been due to the higher biotransformation ability and faster detoxification of benzene in females than in males. Furthermore, the present study observed stronger hematological effects of BTX in overweight subjects. Lipophilic BTX mainly accumulate in fat-enriched tissues. Therefore, fat content in the body might impact their distribution, metabolism, and eventually their toxicity [3]. Taken together, the observed effect modification provided potential evidence for risk assessment. However, further research is needed to determine the underlying mechanisms.

In addition, we found that BTX components had positive combined effects on the decline in monocyte counts, RBC counts, and hemoglobin concentrations. Their combined toxicities have been a great concern for a long time [8]. Previous studies showed that higher levels of toluene inhibited benzene metabolism and subsequent myelotoxicity [34], whereas lower levels of toluene enhanced benzene genotoxicity and confounded its effects on metabolic enzymes and blood cell populations [10,35]. Competitive metabolic interactions are the most plausible mechanisms of BTX combined effects, as BTX are known substrates for CYP2E1 [8]. At high BTX co-exposure levels, the induction of CYP2E1 of a single component might be prohibited by others competitively, while at lower co-exposure levels, CYP2E1 induction causes more BTX components to be metabolized to toxic metabolites [35]. The combined effects of BTX on the decline in hematologic parameters were also observed in our participants, who were co-exposed to lower concentrations of BTX components. Although the underlying mechanisms remain unclear, our study implied that it is necessary to consider the co-exposure effects of BTX components in risk assessment. 

The BMD estimation method is widely used to determine the POD for a specific dose–response relationship, which derives health-based guidance values [36]. The determination of endpoints is the first step for BMD calculations. However, there are currently no standard definitions for hematological damage. Due to the inconsistent findings in studies of BTX-induced hematological effects [15,32,37], it is hard to decide which hematologic parameter can define hematological damage. In addition, using one parameter (such as WBC counts) to define hematological damage would exclude the endpoints, which may be the most sensitive effect in risk assessment using BMD analyses [16]. Thus, it might be more appropriate to define hematological damage based on multiple hematologic parameters, not only one parameter. As a large body of evidence shows that the main hematological effects of BTX (especially benzene) are hematopoietic suppression [2], we considered it hematological damage if the levels of at least two parameters in the follow-up stage were below the lower limits (not the upper limits) of the normal range for Chinese adults. Since we observed significant declines in many hematologic parameters at the four-year follow-up and their magnitude showed sex differences, we also considered it hematological damage if the magnitude of the decline in at least two parameters was higher than the 95th percentile of the population of the same gender.

Based on the calculated BMDLs and up to 40 years of working duration, we proposed 8h-RELs of about 0.01 ppm for BTX components, which were much lower than the current regulatory or recommended exposure limits, such as the Permissible Exposure Limits-TWA (PELs-TWA) developed by U.S. Occupational Safety and Health Administration (OSHA), the Permissible Concentrations-TWA (PCs-TWA) set in China, and the RfCs established by the EPA. Zhang et al. [38] estimated a BMDL for benzene CE levels of about 4.37 mg/m^3^ × year with BMR levels at 10% excess risk of reduced WBC counts, which is higher than our estimated BMDL for benzene (1.559 mg/m^3^ × year). This difference might be attributed to different definitions of hematological damage. However, exposure standards are determined by a trade-off or balancing of the assessed risks and costs for reducing exposure levels. Further research is needed to evaluate the feasibility of our estimated 8h-RELs of BTX components. 

The present study had several major strengths. Firstly, our participants were petrochemical workers regularly exposed to BTX-rich emissions for more than one year, with their occupational BTX sources and levels showing few fluctuations. Thus, BTX CE levels could provide a more accurate estimation of long-term exposure than internal exposure biomarkers mostly reflecting recent exposure. Secondly, we used the BMD estimation method to develop the RELs of BTX exposure based on comprehensive criteria of hematological damage. However, there were limitations in selecting our participants, whereby the participants had no history of diseases or any obvious abnormities at the baseline physical examination that may cause healthy worker selection. Secondly, the present study was conducted on an occupational population, and it is uncertain whether our findings could be extrapolated to the general population. Moreover, although our participants were intentionally selected from workplaces where BTX were the primary occupational hazards, they may also have been co-exposed to unidentified hemotoxins in working and living environments. Despite the adjustment for factory location and lifestyle in the statistical analyses to control their confounding effects, further studies are still warranted to evaluate their influences on our results.

## 5. Conclusions

This follow-up study on BTX-exposed petrochemical workers observed significant single and combined effects of BTX components on the decline in hematologic parameters, providing further perspective evidence of complex hematological effects of long-term occupational exposure to BTX components. The RELs of BTX components based on their hematological effects were established in the present study. Further investigations are needed to determine the underlying mechanisms of hematological effects induced by BTX and assess the feasibility of the 8h-RELs of BTX components estimated in the present study.

## Figures and Tables

**Table 1:** General characteristics, BTX CE levels, and hematologic parameters and their decline at the four-year follow-up.

Variables ^a^	Baseline Stage	Follow-Up Stage	*p*-Value ^b^	Decline in Hematologic Parameters ^c^
**General characteristics**				
Age (years)	39.91 ± 6.57	-	-	-
Sex (male (%))	816 (77.42)	-	-	-
Smoking status (smoker (%))	343 (32.54)	-	-	-
Drinking status (drinker (%))	493 (46.77)	-	-	-
Factory location (Guangzhou (%))	387 (36.72)	-	-	-
BMI (kg/m^3^)	23.33 ± 3.11	-	-	-
Pack-years of smoking	2.84 ± 6.13	-	-	-
Years of occupational exposure (years)	18.75 ± 7.70	-	-	-
**BTX CE levels (mg/m^3^ × year)**				
Benzene	0.65 (0.36, 1.03)	-	-	-
Toluene	0.84 (0.56, 1.23)	-	-	-
Xylene	1.37 (0.71, 2.40)	-	-	-
**Hematologic parameters**				
WBCs ( × 10^9^/L)	6.49 ± 1.58	6.41 ± 2.47	0.287	0.08 ± 2.35
Neutrophils ( × 10^9^/L)	3.54 ± 1.15	3.57 ± 1.17	0.293	−0.03 ± 1.03
Monocytes ( × 10^9^/L)	0.47 ± 0.23	0.32 ± 0.12	<0.001	0.15 ± 0.22
Lymphocytes ( × 10^9^/L)	2.42 ± 0.67	2.27 ± 0.65	<0.001	0.15 ± 0.54
RBCs ( × 10^12^/L)	5.05 ± 0.60	4.97 ± 0.58	<0.001	0.08 ± 0.39
Hemoglobin (g/L)	152.10 ± 17.93	143.76 ± 14.69	<0.001	8.34 ± 13.85
Hematocrit (%)	43.76 ± 4.88	43.62 ± 3.87	0.256	0.14 ± 3.90
Platelets ( × 10^9^/L)	243.60 ± 58.01	241.90 ± 54.67	0.152	1.70 ± 38.07
MPV (fL)	9.52 ± 1.18	9.03 ± 1.40	<0.001	0.49 ± 0.74

Note: Abbreviations: BMI, body mass index; CE, cumulative exposure; WBC, white blood cell; RBC, red blood cell; MPV, mean platelet volume. ^a^ Mean ± standard deviation, n (%), or median (25th percentile, 75th percentile). ^b^ Paired Student’s *t*-test. ^c^ The decline in hematologic parameters was defined as the levels measured in the baseline stage minus the corresponding levels measured in the follow-up stage.

**Table 2:** Associations between the CE levels of individual BTX components and the decline in hematologic parameters.

Decline in Hematologic Parameters	Benzene		Toluene		Xylene	
	β (95%CI)	*P*_trend_ ^a^	β (95%CI)	*P*_trend_ ^a^	β (95%CI)	*P*_trend_ ^a^
WBCs	0.047 (−0.127, 0.221)	0.596	0.018 (−0.170, 0.206)	0.855	0.025 (−0.200, 0.250)	0.825
Neutrophils	0.011 (−0.062, 0.084)	0.761	−0.029 (−0.107, 0.049)	0.460	−0.027 (−0.121, 0.067)	0.572
Monocytes	0.012 (0.004, 0.020)	0.007	0.003 (−0.007, 0.013)	0.478	0.005 (−0.007, 0.017)	0.368
Lymphocytes	0.031 (−0.006, 0.068)	0.104	0.047 (0.008, 0.086)	0.021	0.041 (−0.006, 0.088)	0.091
RBCs	−0.004 (−0.026, 0.018)	0.698	0.010 (−0.014, 0.034)	0.376	0.014 (−0.013, 0.041)	0.315
Hemoglobin	0.093 (−0.526, 0.712)	0.769	0.202 (−0.470, 0.874)	0.556	0.436 (−0.360, 1.232)	0.282
Hematocrit	0.137 (−0.045, 0.319)	0.143	0.189 (−0.009, 0.387)	0.061	0.259 (0.022, 0.496)	0.032
Platelets	1.401 (−1.198, 4.000)	0.291	−0.021 (−2.841, 2.799)	0.988	0.935 (−2.430, 4.300)	0.586
MPV	0.002 (−0.041, 0.045)	0.927	0.009 (−0.038, 0.056)	0.723	0.026 (−0.031, 0.083)	0.362

Note: Abbreviations: WBC, white blood cell; RBC, red blood cell; MPV, mean platelet volume. ^a^ Generalized linear model with adjustment for age, sex, factory location, BMI, smoking status, pack-years of smoking, drinking status, and baseline hematologic parameters.

**Table 3:** Significant ^a^ modification effects of baseline parameters and general characteristics on the associations of BTX CE levels with decline in hematologic parameters.

BTX Component	Decline in Hematologic Parameters	Associations (β (95% CI)) ^b^ in Different Subgroups			*P*_interaction_^c^
**Stratified by baseline parameters ^d^**		**Low**	**Medium**	**High**	
Benzene	Monocytes	0.015 (0.001, 0.029) ^§^	0.004 (−0.010, 0.018)	0.026 (−0.009, 0.061)	0.008
Toluene	Monocytes	0.009 (−0.005, 0.023)	1.98 × 10^−4^ (−0.015, 0.016)	0.021 (−0.020, 0.062)	<0.001
Xylene	Monocytes	0.011 (−0.007, 0.029)	−0.006 (−0.024, 0.012)	0.053 (0.004, 0.102) ^§^	<0.001
Toluene	RBC	−0.012 (−0.045, 0.021)	0.016 (−0.023, 0.055)	0.059 (0.006, 0.112) ^§^	0.027
Xylene	RBC	−4.90 × 10^−4^ (−0.042, 0.041)	2.96 × 10^−4^ (−0.047, 0.047)	0.085 (0.028, 0.142) ^§^	<0.001
Toluene	Hemoglobin	−0.292 (−1.515, 0.931)	0.201 (−0.779, 1.181)	1.647 (0.120, 3.174) ^§^	0.012
Toluene	Hematocrit	0.153 (−0.186, 0.492)	0.006 (−0.286, 0.298)	0.555 (0.063, 1.047) ^§^	0.014
Xylene	Hematocrit	0.235 (−0.184, 0.654)	−0.030 (−0.387, 0.327)	0.780 (0.247, 1.313) ^§^	0.001
**Stratified by sex**		**Female**	**Male**		
Xylene	Lymphocytes	0.044 (−0.032, 0.120)	0.061 (0.002, 0.120) ^§^		0.001
Toluene	RBC	−0.021 (−0.080, 0.038)	0.018 (−0.007, 0.043)		0.038
Xylene	RBC	−0.026 (−0.081, 0.029)	0.032 (0.001, 0.063) ^§^		0.001
Xylene	Hemoglobin	−0.751 (−2.640, 1.138)	1.003 (0.125, 1.881) ^§^		0.036
Benzene	Hematocrit	−0.347 (−0.917, 0.223)	0.206 (0.022, 0.390) ^§^		0.033
Toluene	Hematocrit	−0.420 (−1.067, 0.227)	0.271 (0.073, 0.469) ^§^		0.011
Xylene	Hematocrit	−0.249 (−0.870, 0.372)	0.401 (0.150, 0.652) ^§^		0.033
**Stratified by drinking status**		**Nondrinker**	**Drinker**		
Benzene	Neutrophils	0.069 (−0.031, 0.169)	−0.044 (−0.152, 0.064)		0.048
Benzene	Lymphocytes	0.045 (−0.006, 0.096)	0.025 (−0.030, 0.080)		0.030
Toluene	Lymphocytes	0.068 (0.013, 0.123) ^§^	0.026 (−0.033, 0.085)		0.033
**Stratified by BMI (kg/m^2^)**		**<24**	**≥24**		
Benzene	Neutrophils	−0.017 (−0.111, 0.077)	0.065 (−0.049, 0.179)		0.012
Toluene	Neutrophils	−0.101 (−0.205, 0.003)	0.070 (−0.050, 0.190)		0.001
Toluene	Lymphocytes	0.016 (−0.037, 0.069)	0.086 (0.023, 0.149) ^§^		0.029
Xylene	Lymphocytes	0.010 (−0.049, 0.069)	0.093 (0.011, 0.175)^§^		0.023

Note: Abbreviations: RBC, red blood cell. ^a^ This table only shows significant modification effects that had a *P*_interaction_ lower than 0.05. ^b^ Generalized linear model with adjustment for age, sex, factory location, BMI, smoking status, pack-years of smoking, drinking status, and/or baseline hematologic parameters when appropriate. ^c^ *P*_interaction_ was calculated by modeling an interaction term of each BTX component and each stratified variable in generalized linear models, with adjustment for age, sex, factory location, BMI, smoking status, pack-years of smoking, drinking status, and/or baseline hematologic parameters when appropriate. ^d^ Subjects were divided into three groups (low, medium, and high) according to the tertiles of the baseline hematologic parameters. **^§^** *P*_trend_ < 0.05.

**Table 4:** Associations of the WQS regression indices with the decline in hematologic parameters.

Decline in Hematologic Parameters	WQS Index	β (95% CI) for WQS Index ^a^	*P*_trend_ ^a^	Weight for Each BTX Component ^b^		
				Benzene	Toluene	Xylene
WBCs	WQS_WBC_	0.175 (−0.107, 0.457)	0.226	0.677	0.099	0.224
Neutrophils	WQS_NEUT_	0.008 (−0.096, 0.112)	0.878	0.26	0.425	0.315
Monocytes	WQS_MOE_	0.013 (0.003, 0.023)	0.007	0.976	0	0.024
Lymphocytes	WQS_LYM_	0.005 (−0.050, 0.060)	0.851	0.038	0.303	0.659
RBCs	WQS_RBC_	0.045 (0.018, 0.072)	0.002	0	0.614	0.386
Hemoglobin	WQS_Hb_	0.972 (0.045, 1.899)	0.040	0.073	0.08	0.847
Hematocrit	WQS_HCT_	0.162 (−0.112, 0.436)	0.248	0.009	0.153	0.838
Platelets	WQS_PLT_	0.147 (−3.810, 4.104)	0.942	0.049	0.443	0.508
MPV	WQS_MPV_	−0.032 (−0.091, 0.027)	0.282	0.078	0.498	0.424

Note: Abbreviations: WQS, weighted quantile sum; WBC, white blood cell; RBC, red blood cell; MPV, mean platelet volume. ^a^ Estimated β was associated with a quartile increase in the WQS index that was calculated using WQS regression models with adjustment for age, sex, factory location, BMI, smoking status, pack-years of smoking, drinking status, and the corresponding baseline hematologic parameters. ^b^ The gray scale reflects the magnitude of weights; the deeper the color is, the higher the weight.

**Table 5:** Estimated BMDs and BMDLs for BTX components based on their hematological effects.

BTX Component	Model Name	Chi-Squared	*p* for Goodness-of-Fit Tests	AIC	BMD (mg/m^3^ × year)	BMDL (mg/m^3^ × year)
Benzene	Multistage ^a^	6.017	0.421	777.588	2.138	1.559
Toluene	Dichotomous Hill ^b^	4.322	0.364	704.276	1.449	1.325
Xylene	Probit ^c^	5.669	0.461	741.547	2.937	2.312

Note: Abbreviations: AIC, Akaike’s Information Criterion; BMD, benchmark dose; BMDL, lower confidence limit of BMD. ^a^ The Multistage dose–response model was represented as *P*[X] = background + (1 − background) × [1 − exp(−slope_1_ × X_1_ − slope_2_ × X_2_−…)]. ^b^ The Dichotomous Hill dose–response model was defined by *P*[X] = background + (v − v × background)/[1 + exp(−intercept − slope × Log(X))], where v is the maximum probability of response predicted by the model. ^c^ The Probit dose–response model was represented as *P*[X] = CumNorm(intercept + slope × X).

## Data Availability

The data presented in this study are available upon request from the corresponding authors. The data are not publicly available due to the regulations of the authors’ affiliations.

## Supplementary Materials

The following supporting information can be downloaded at: https://www.mdpi.com/article/10.3390/toxics10090502/s1, Table S1: Information on ambient BTX monitoring in workplaces three years before the baseline stage, Table S2: Normal range of hematologic parameters according to WS/T 405-2012, Table S3: Incidence proportion of hematological damage in subgroups with different BTX CE levels, Table S4: Correlation of 8h-TWA concentrations of BTX components, Table S5: Decline in hematologic parameters in subjects with different general characteristics, Table S6: Associations of BTX CE levels with decline in hematologic parameters stratified by corresponding baseline hematologic parameters, Table S7: Associations of BTX CE levels with decline in hematologic parameters stratified by age, Table S8: Associations of BTX CE levels with decline in hematologic parameters stratified by sex, Table S9: Associations of BTX CE levels with decline in hematologic parameters stratified by smoking status, Table S10: Associations of BTX CE levels with decline in hematologic parameters stratified by drinking status, Table S11: Associations of BTX CE levels with decline in hematologic parameters stratified by BMI (kg/m^2^), Figure S1: Comparisons of annual average BTX 8h-TWA concentrations using generalized estimating equation models, Figure S2: Dose–response curves and estimated BMDs and BMDLs of CE levels of benzene (A), toluene (B), and xylene (C) for the incidence proportions of hematological damage.
