# Supplementary material for: Hematological Effects and Benchmark Doses of Long-Term Co-Exposure to Benzene, Toluene, and Xylenes in a Follow-Up Study on Petrochemical Workers
Source: Toxics. 2022 Aug 28;10(9):502. doi: 10.3390/toxics10090502 (PMC9501893; doi:10.3390/toxics10090502)
Supplement: Supplementary file 1 [file toxics-10-00502-s001.zip › toxics-1861216-supplementary.pdf]

Supplementary material

Hematological Effects and Benchmark Doses of Long-Term Co-Exposure to Benzene, Toluene, and Xylenes in a Follow-Up Study on Petrochemical Workers

Zhaorui Zhang†, Xin Liu†, Chaofan Guo, Xinjie Zhang, Yingying Zhang, Na Deng, Guanchao Lai, Aichu Yang, Yongshun Huang, Shanfeng Dang, Yanqun Zhu, Xiumei Xing, Yongmei Xiao‡, and Qifei Deng‡

†These authors contributed equally to this work.

‡These authors jointly supervised this work.

Table S1. Information for ambient BTX monitoring in workplaces in three years before the baseline stage.

Number of equipment releasing BTX in a workplace	Number of workplaces	Number of sampling point(s) for each workplace [a]	Sampling frequency during three years	Total number of duplicate air samples	No. (%) < LOD [b]		
					Benzene	Toluene	Xylene
1-3	22	1	4 seasons×1.5 years for one newly constructed workplace; 4 seasons ×3 years for others	258	24 (9.30)	21 (8.14)	19 (7.36)
4-10	9	2	4 seasons ×3 years	216	10 (4.63)	11 (5.09)	7 (3.24)
>10	1	3	4 seasons ×3 years	36	2 (5.56)	1 (2.78)	1 (2.78)
Total	32			510	36 (7.06)	33 (6.47)	27 (5.29)

Abbreviations: LOD, limit of detection.

[a] If there are more than 10 equipment releasing BTX components in a workplace, 3 sampling points were set up for each workplace; if there are 4-10 equipment releasing BTX components, 2 sampling points were set up; and if there are 1-3 equipment releasing BTX components, 1 sampling point weas set up.

[b] Number (percentage) of samples with concentrations below LOD;

Table S2. Normal range of hematologic parameters according to WS/T 405-2012.

Hematologic parameters	Normal range in Chinese adults
WBC counts	3.5-9.5 $\times 10^9$/L
Neutrophil counts	1.8-6.3 $\times 10^9$/L
Monocyte counts	0.1-0.6 $\times 10^9$/L
Lymphocyte counts	1.1-3.2 $\times 10^9$/L
RBC counts	Male: 4.3-5.8$\times 10^{12}$/L; Female: 3.8-5.1$\times 10^{12}$/L
Hemoglobin	Male: 130-175 g/L; Female: 115-150 g/L
Hematocrit	Male: 40%-50%; Female: 35%-45%
Platelet counts	120-350 $\times 10^9$/L
MPV	None

Table S3. Incidence proportion of hematological damages in subgroups with different BTX CE levels.

CE ranges (mg/m^3×year)	Median CE levels (mg/m^3×year)	n	Number of cases	Incidence proportion (%)
**Benzene**				
<0.25	0.142	126	17	13.49
0.25~0.40	0.327	156	28	17.95
0.40-0.525	0.477	122	18	14.75
0.525-0.75	0.637	186	28	15.05
0.75-0.90	0.831	120	24	20.00
0.90-1.20	1.050	180	32	17.78
1.20-2.00	1.341	93	11	11.83
>2.00	2.551	71	13	18.31
**Toluene**				
<0.30	0.161	146	12	8.22
0.30-0.60	0.470	140	19	13.57
0.60-0.80	0.662	199	39	19.60
0.80-1.10	0.929	192	32	16.67
1.10-1.40	1.210	189	26	13.76
1.40-1.90	1.580	114	32	28.07
>1.90	2.470	74	11	14.86
**Xylene**				

<0.30	0.164	95	10	10.53
0.30-0.60	0.420	122	8	6.56
0.60-0.80	0.700	92	7	7.61
0.80-1.10	0.875	123	12	9.76
1.10-1.60	1.342	173	24	13.87
1.60-2.10	1.861	144	29	20.14
2.10-3.10	2.561	142	34	23.94
>3.10	3.582	163	47	28.83

Table S4. The correlation [a] of 8h-TWA concentrations of BTX components.

BTX components	Toluene	Xylene
Benzene	0.502**	0.445**
Toluene	-	0.440**

[a] Spearman' s rank correlation test;

** $P< 0.001$.

Table S5. Decline in hematologic parameters in subjects with different general characteristics.

Decline in hematologic parameters [a]	Age		Sex		Smoking status		Drinking status		BMI	
	≤40 years (n=573)	>40 years (n=481)	Female (n=238)	Male (n=816)	Nonsmoker (n=711)	Smoker (n=343)	Nondrinker (n=561)	Drinker (n=493)	<24 kg/m² (n=646)	≥24 kg/m² (n=408)
WBC	4.0×10⁻³±2.88	0.18±1.39	0.18±1.22	0.05±2.58	0.05±2.63	0.13±1.57	0.13±1.37	0.02±3.12	0.02±2.82	0.17±1.34
Neutrophils	-0.04±1.08	-0.03±0.97	-0.03±1.00	-0.04±1.04	-0.04±1.00	-0.03±1.09*	-0.01±1.03	-0.06±1.03	-0.04±1.01	-0.02±1.06
Monocytes	0.12±0.18	0.18±0.25	0.12±0.17	0.16±0.23*	0.13±0.20	0.19±0.25**	0.14±0.21	0.16±0.22	0.14±0.21	0.17±0.22
Lymphocytes	0.10±0.54	0.20±0.54*	0.18±0.41	0.14±0.57**	0.13±0.52	0.18±0.57*	0.14±0.52	0.15±0.56	0.16±0.53	0.13±0.56*
RBC	0.01±0.38	0.16±0.38	0.01±0.35	0.10±0.40	0.07±0.39	0.10±0.39	0.07±0.40	0.08±0.37	0.08±0.39	0.08±0.39
Hemoglobin	6.17±13.92	11.02±13.29	6.50±13.52	8.88±13.90**	8.09±13.80	8.88±13.95	8.36±14.05	8.31±13.63	8.14±13.74	8.64±14.01
Hematocrit	-0.54±3.61	1.00±4.10	-0.07±4.43	0.20±3.74**	0.14±4.02	0.13±3.65	0.21±4.15	0.06±3.60	0.19±3.98	0.06±3.79*
Platelet	-0.82±38.22	4.94±37.66	-2.69±38.28	2.98±37.93**	0.73±38.54	3.76±37.01	2.10±39.20	1.24±36.75	1.43±39.69	2.12±35.49
MPV	0.41±0.70	0.60±0.78	0.46±0.70	0.51±0.75	0.51±0.75	0.47±0.72	0.49±0.71	0.51±0.77	0.51±0.77	0.48±0.70

Note: Abbreviation: WBC, white blood cell; RBC, red blood cell; MPV, mean platelet volume.

[a] Values of variables were shown by mean± SD.

The between-group differences in decline in hematologic parameters were analyzed by multivariable covariance analysis, with adjustment for age, sex, factory location, BMI, smoking status, pack-years of smoking, drinking status, and/or the corresponding baseline hematologic parameters, when appropriate.

* *P*< 0.05; ** *P*< 0.001.

Table S6. Associations of BTX CE levels with decline in hematologic parameters stratified by the corresponding baseline hematologic parameters.

Decline in hematologic parameters	Groups of baseline parameters[a]	Benzene			Toluene			Xylene		
		β (95% CI)	P_{trend}[b]	$P_{interaction}$[c]	β (95% CI)	P_{trend}[b]	$P_{interaction}$[c]	β (95% CI)	P_{trend}[b]	$P_{interaction}$[c]
WBC	Low	-0.027 (-0.156, 0.102)	0.677	0.360	-0.088 (-0.239, 0.063)	0.254	0.383	-0.083 (-0.254, 0.088)	0.336	0.741
	Medium	0.161 (-0.388, 0.710)	0.566		0.128 (-0.417, 0.673)	0.644		0.132 (-0.550, 0.814)	0.704	
	High	-0.003 (-0.199, 0.193)	0.974		-0.032 (-0.240, 0.176)	0.762		0.037 (-0.212, 0.286)	0.769	
Neutrophils	Low	0.029 (-0.079, 0.137)	0.591	0.632	-0.013 (-0.127, 0.101)	0.828	0.744	-0.036 (-0.167, 0.095)	0.591	0.891
	Medium	0.016 (-0.076, 0.108)	0.736		0.017 (-0.089, 0.123)	0.744		0.017 (-0.114, 0.148)	0.800	
	High	-0.047 (-0.251, 0.157)	0.651		-0.098 (-0.304, 0.108)	0.348		-0.074 (-0.319, 0.171)	0.552	
Monocytes	Low	0.015 (0.001, 0.029)	0.034	0.008	0.009 (-0.005, 0.023)	0.235	8.70×10^{-5}	0.011 (-0.007, 0.029)	0.226	2.56×10^{-9}
	Medium	0.004 (-0.010, 0.018)	0.599		1.98×10^{-4} (-0.015, 0.016)	0.980		-0.006 (-0.024, 0.012)	0.517	
	High	0.026 (-0.009, 0.061)	0.152		0.021 (-0.020, 0.062)	0.320		0.053 (0.004, 0.102)	0.034	
Lymphocytes	Low	0.035 (-0.022, 0.092)	0.219	0.654	0.085 (0.022, 0.148)	0.009	0.863	0.069 (-0.004, 0.142)	0.066	0.179
	Medium	0.037 (-0.026, 0.100)	0.255		0.025 (-0.046, 0.096)	0.500		0.049 (-0.037, 0.135)	0.264	
	High	0.020 (-0.058, 0.098)	0.622		0.052 (-0.026, 0.130)	0.193		0.022 (-0.074, 0.118)	0.650	
RBC	Low	-0.025 (-0.058, 0.008)	0.137	0.328	-0.012 (-0.045, 0.021)	0.490	0.027	-4.90×10^{-4} (-0.042, 0.041)	0.981	4.96×10^{-5}
	Medium	-0.002 (-0.039, 0.035)	0.919		0.016 (-0.023, 0.055)	0.438		2.96×10^{-4} (-0.047, 0.047)	0.990	
	High	0.036 (-0.005, 0.077)	0.084		0.059 (0.006, 0.112)	0.028		0.085 (0.028, 0.142)	0.003	
Hemoglobin	Low	-0.036 (-1.281, 1.209)	0.955	0.138	-0.292 (-1.515, 0.931)	0.640	0.012	0.024 (-1.471, 1.519)	0.975	0.068
	Medium	0.060 (-0.847, 0.967)	0.897		0.201 (-0.779, 1.181)	0.687		0.426 (-0.732, 1.584)	0.472	
	High	0.577 (-0.638, 1.792)	0.353		1.647 (0.120, 3.174)	0.035		1.524 (-0.215, 3.263)	0.087	
Hematocrit	Low	0.140 (-0.219, 0.499)	0.443	0.100	0.153 (-0.186, 0.492)	0.378	0.014	0.235 (-0.184, 0.654)	0.272	0.001
	Medium	-0.042 (-0.311, 0.227)	0.757		0.006 (-0.286, 0.298)	0.970		-0.030 (-0.387, 0.327)	0.868	
	High	0.406 (0.032, 0.780)	0.034		0.555 (0.063, 1.047)	0.028		0.780 (0.247, 1.313)	0.004	
Platelet	Low	1.801 (-2.272, 5.874)	0.387	0.195	-0.486 (-4.776, 3.804)	0.825	0.482	0.947 (-4.351, 6.245)	0.726	0.770
	Medium	2.148 (-1.568, 5.864)	0.258		-0.961 (-4.955, 3.033)	0.638		2.085 (-2.445, 6.615)	0.368	
	High	-0.164 (-6.011, 5.683)	0.956		-0.788 (-7.438, 5.862)	0.816		-1.472 (-9.522, 6.578)	0.720	
MPV	Low	-0.073 (-0.151, 0.005)	0.067	0.090	-0.017 (-0.123, 0.089)	0.754	0.220	0.035 (-0.075, 0.145)	0.529	0.617
	Medium	0.024 (-0.052, 0.100)	0.533		0.015 (-0.065, 0.095)	0.720		0.042 (-0.054, 0.138)	0.396	
	High	0.023 (-0.067, 0.113)	0.609		0.005 (-0.083, 0.093)	0.909		0.001 (-0.111, 0.113)	0.983	

Note: Abbreviation: WBC, white blood cell; RBC, red blood cell; MPV, mean platelet volume.

[a] Subjects were divided to three groups (low, medium, and high) groups according to the tertile of the baseline hematologic parameters.

[b] Generalized linear model with adjustment for age, sex, factory location, BMI, smoking status, pack-years of smoking, and drinking status;

[c] $P_{interaction}$ were calculated by modeling an interaction term of single BTX components and baseline hematologic parameters in generalized linear models, with adjustment for age, sex, factory location, BMI, smoking status, pack-years of smoking, and drinking status.

Table S7. Associations of BTX CE levels with decline in hematologic parameters stratified by age.

Decline in hematologic parameters	Age group	Benzene			Toluene			Xylene		
		β (95% CI)	P_{trend}[a]	$P_{interaction}$[b]	β (95% CI)	P_{trend}[a]	$P_{interaction}$[b]	β (95% CI)	P_{trend}[a]	$P_{interaction}$[b]
WBC	≤40	0.050 (-0.171, 0.271)	0.654	0.892	0.052 (-0.166, 0.270)	0.639	0.602	0.101 (-0.164, 0.366)	0.452	0.436
	>40	0.128 (-0.066, 0.322)	0.196		-0.041 (-0.274, 0.192)	0.729		-0.198 (-0.433, 0.037)	0.101	
Neutrophils	≤40	-0.037 (-0.113, 0.039)	0.345	0.089	-0.064 (-0.138, 0.010)	0.095	0.064	-0.066 (-0.158, 0.026)	0.159	0.126
	>40	0.139 (-1.60×10^{-4}, 0.278)	0.050		0.138 (-0.029, 0.305)	0.104		0.107 (-0.064, 0.278)	0.218	
Monocytes	≤40	0.004 (-0.004, 0.012)	0.342	0.050	-4.54×10^{-4} (-0.008, 0.007)	0.912	0.371	0.001 (-0.009, 0.011)	0.908	0.687
	>40	0.023 (0.005, 0.041)	0.010		0.008 (-0.014, 0.030)	0.459		0.006 (-0.016, 0.028)	0.563	
Lymphocytes	≤40	0.043 (0.006, 0.080)	0.025	0.704	0.056 (0.019, 0.093)	0.003	0.764	0.075 (0.030, 0.120)	0.001	0.300
	>40	0.066 (-0.010, 0.142)	0.088		0.079 (-0.013, 0.171)	0.095		0.009 (-0.085, 0.103)	0.850	
RBC	≤40	0.009 (-0.011, 0.029)	0.381	0.182	0.018 (-0.002, 0.038)	0.073	0.218	0.016 (-0.008, 0.040)	0.200	0.965
	>40	-0.018 (-0.061, 0.025)	0.410		-0.005 (-0.058, 0.048)	0.856		0.031 (-0.024, 0.086)	0.268	
Hemoglobin	≤40	-0.201 (-0.834, 0.432)	0.534	0.607	-0.051 (-0.674, 0.572)	0.874	0.653	-0.042 (-0.801, 0.717)	0.913	0.133
	>40	0.549 (-0.672, 1.770)	0.379		0.525 (-0.961, 2.011)	0.489		0.946 (-0.557, 2.449)	0.218	
Hematocrit	≤40	0.082 (-0.083, 0.247)	0.331	0.854	0.122 (-0.041, 0.285)	0.141	0.898	0.108 (-0.090, 0.306)	0.286	0.108
	>40	0.257 (-0.155, 0.669)	0.222		0.268 (-0.236, 0.772)	0.298		0.510 (4.00×10^{-4}, 1.020)	0.050	
Platelet	≤40	0.613 (-1.862, 3.088)	0.628	0.369	0.030 (-2.412, 2.472)	0.981	0.844	0.844 (-2.125, 3.813)	0.578	0.505
	>40	3.081 (-2.548, 8.710)	0.284		-1.117 (-7.995, 5.761)	0.750		-0.699 (-7.675, 6.277)	0.844	
MPV	≤40	-0.003 (-0.046, 0.040)	0.875	0.257	-0.010 (-0.051, 0.031)	0.632	0.714	-0.018 (-0.069, 0.033)	0.487	0.070
	>40	-0.044 (-0.138, 0.050)	0.362		0.020 (-0.096, 0.136)	0.730		0.086 (-0.032, 0.204)	0.152	

Note: Abbreviation: WBC, white blood cell; RBC, red blood cell; MPV, mean platelet volume.

[a] Generalized linear model with adjustment for sex, factory location, BMI, smoking status, pack-years of smoking, drinking status, and the corresponding baseline hematologic parameters;

[b] $P_{interaction}$ were calculated by modeling an interaction term of single BTX components and age groups in generalized linear models, with adjustment for sex, factory location, BMI, smoking status, pack-years of smoking, drinking status, and the corresponding baseline hematologic parameters.

Table S8. Associations of BTX CE levels with decline in hematologic parameters stratified by sex.

Decline in hematologic parameters	Sex	Benzene			Toluene			Xylene		
		β (95% CI)	P_{trend}[a]	$P_{interaction}$[b]	β (95% CI)	P_{trend}[a]	$P_{interaction}$[b]	β (95% CI)	P_{trend}[a]	$P_{interaction}$[b]
WBC	Female	0.150 (-0.052, 0.352)	0.145	0.587	0.143 (-0.086, 0.372)	0.223	0.802	0.073 (-0.148, 0.294)	0.520	0.480
	Male	0.040 (-0.174, 0.254)	0.714		0.004 (-0.223, 0.231)	0.975		0.033 (-0.253, 0.319)	0.819	
Neutrophils	Female	0.078 (-0.091, 0.247)	0.366	0.668	0.002 (-0.190, 0.194)	0.987	0.601	-0.013 (-0.195, 0.169)	0.887	0.160
	Male	0.008 (-0.074, 0.090)	0.843		-0.025 (-0.111, 0.061)	0.566		-0.011 (-0.121, 0.099)	0.845	
Monocytes	Female	0.020 (4.00×10^{-4}, 0.040)	0.042	0.818	0.017 (-0.005, 0.039)	0.126	0.627	0.008 (-0.014, 0.030)	0.437	0.815
	Male	0.010 (2.00×10^{-4}, 0.020)	0.042		0.001 (-0.009, 0.011)	0.868		0.004 (-0.010, 0.018)	0.586	
Lymphocytes	Female	0.036 (-0.035, 0.107)	0.309	0.618	0.059 (-0.019, 0.137)	0.145	0.482	0.044 (-0.032, 0.120)	0.254	0.001
	Male	0.041 (-0.002, 0.084)	0.064		0.052 (0.007, 0.097)	0.026		0.061 (0.002, 0.120)	0.041	
RBC	Female	-0.035 (-0.086, 0.016)	0.175	0.145	-0.021 (-0.080, 0.038)	0.471	0.038	-0.026 (-0.081, 0.029)	0.359	0.001
	Male	0.005 (-0.019, 0.029)	0.680		0.018 (-0.007, 0.043)	0.155		0.032 (0.001, 0.063)	0.044	
Hemoglobin	Female	-0.931 (-2.652, 0.790)	0.290	0.135	-1.031 (-3.001, 0.939)	0.306	0.080	-0.751 (-2.640, 1.138)	0.437	0.036
	Male	0.405 (-0.252, 1.062)	0.227		0.523 (-0.179, 1.225)	0.145		1.003 (0.125, 1.881)	0.026	
Hematocrit	Female	-0.347 (-0.917, 0.223)	0.235	0.033	-0.420 (-1.067, 0.227)	0.204	0.011	-0.249 (-0.870, 0.372)	0.434	0.033
	Male	0.206 (0.022, 0.390)	0.029		0.271 (0.073, 0.469)	0.007		0.401 (0.150, 0.652)	0.002	
Platelet	Female	-3.061 (-9.533, 3.411)	0.355	0.059	-2.159 (-9.546, 5.228)	0.567	0.170	-2.953 (-10.033, 4.127)	0.414	0.082
	Male	2.553 (-0.332, 5.438)	0.083		0.556 (-2.527, 3.639)	0.724		2.595 (-1.317, 6.507)	0.194	
MPV	Female	0.043 (-0.061, 0.147)	0.414	0.494	-0.023 (-0.141, 0.095)	0.702	0.587	0.047 (-0.065, 0.159)	0.414	0.913
	Male	-0.005 (-0.054, 0.044)	0.831		0.014 (-0.039, 0.067)	0.600		0.022 (-0.045, 0.089)	0.517	

Note: Abbreviation: WBC, white blood cell; RBC, red blood cell; MPV, mean platelet volume.

[a]Generalized linear model with adjustment for age, factory location, BMI, smoking status, pack-years of smoking, drinking status, and the corresponding baseline hematologic parameters;

[b]$P_{interaction}$ were calculated by modeling an interaction term of single BTX components and sex in generalized linear models, with adjustment for age, factory location, BMI, smoking status, pack-years of smoking, drinking status, and the corresponding baseline hematologic parameters.

Table S9. Associations of BTX CE levels with decline in hematologic parameters stratified by smoking status.

Decline in hematologic parameters	Smoking status	Benzene			Toluene			Xylene		
		β (95% CI)	P_{trend}[a]	$P_{interaction}$[b]	β (95% CI)	P_{trend}[a]	$P_{interaction}$[b]	β (95% CI)	P_{trend}[a]	$P_{interaction}$[b]
WBC	Nonsmoker	0.002 (-0.239, 0.243)	0.990	0.991	-0.045 (-0.304, 0.214)	0.733	0.738	-0.022 (-0.326, 0.282)	0.889	0.432
	Smoker	0.171 (-0.031, 0.373)	0.099		0.166 (-0.050, 0.382)	0.131		0.179 (-0.095, 0.453)	0.202	
Neutrophils	Nonsmoker	-0.012 (-0.096, 0.072)	0.779	0.707	-0.064 (-0.154, 0.026)	0.171	0.308	-0.035 (-0.143, 0.073)	0.520	0.169
	Smoker	0.080 (-0.059, 0.219)	0.265		0.050 (-0.099, 0.199)	0.512		0.030 (-0.160, 0.220)	0.761	
Monocytes	Nonsmoker	0.014 (0.004, 0.024)	0.007	0.909	0.007 (-0.003, 0.017)	0.207	0.526	0.004 (-0.008, 0.016)	0.545	0.762
	Smoker	0.008 (-0.008, 0.024)	0.374		-0.004 (-0.022, 0.014)	0.689		0.009 (-0.015, 0.033)	0.425	
Lymphocytes	Nonsmoker	0.029 (-0.016, 0.074)	0.204	0.836	0.046 (-0.001, 0.093)	0.059	0.873	0.036 (-0.021, 0.093)	0.205	0.072
	Smoker	0.053 (-0.018, 0.124)	0.141		0.053 (-0.021, 0.127)	0.163		0.085 (-0.009, 0.179)	0.079	
RBC	Nonsmoker	0.005 (-0.020, 0.030)	0.685	0.181	0.023 (-0.004, 0.050)	0.109	0.241	0.023 (-0.010, 0.056)	0.174	0.826
	Smoker	-0.021 (-0.058, 0.016)	0.258		-0.014 (-0.053, 0.025)	0.485		-0.002 (-0.053, 0.049)	0.923	
Hemoglobin	Nonsmoker	0.103 (-0.654, 0.860)	0.790	0.708	0.225 (-0.594, 1.044)	0.590	0.655	0.485 (-0.466, 1.436)	0.318	0.743
	Smoker	0.054 (-1.065, 1.173)	0.925		0.161 (-1.039, 1.361)	0.792		0.309 (-1.224, 1.842)	0.693	
Hematocrit	Nonsmoker	0.163 (-0.072, 0.398)	0.176	0.948	0.250 (-0.005, 0.505)	0.054	0.716	0.257 (-0.043, 0.557)	0.093	0.640
	Smoker	0.089 (-0.201, 0.379)	0.551		0.053 (-0.259, 0.365)	0.738		0.278 (-0.118, 0.674)	0.171	
Platelet	Nonsmoker	1.300 (-1.873, 4.473)	0.422	0.855	0.357 (-3.077, 3.791)	0.839	0.380	1.120 (-2.922, 5.162)	0.587	0.263
	Smoker	1.318 (-3.366, 6.002)	0.582		-1.298 (-6.300, 3.704)	0.611		-0.735 (-7.109, 5.639)	0.821	
MPV	Nonsmoker	-0.009 (-0.064, 0.046)	0.761	0.633	0.010 (-0.049, 0.069)	0.748	0.388	0.008 (-0.063, 0.079)	0.818	0.264
	Smoker	0.022 (-0.052, 0.096)	0.554		-0.003 (-0.081, 0.075)	0.938		0.064 (-0.036, 0.164)	0.211	

Note: Abbreviation: WBC, white blood cell; RBC, red blood cell; MPV, mean platelet volume.

[a] Generalized linear model with adjustment for age, sex, factory location, BMI, drinking status, and the corresponding baseline hematologic parameters;

[b] $P_{interaction}$ were calculated by modeling an interaction term of single BTX components and smoking status in generalized linear models, with adjustment for age, sex, factory location, BMI, drinking status, and the corresponding baseline hematologic parameters.

Table S10. Associations of BTX CE levels with decline in hematologic parameters stratified by drinking status.

Decline in hematologic parameters	Drinking status	Benzene			Toluene			Xylene		
		β (95% CI)	P_{trend} [a]	$P_{interaction}$ [b]	β (95% CI)	P_{trend} [a]	$P_{interaction}$ [b]	β (95% CI)	P_{trend} [a]	$P_{interaction}$ [b]
WBC	Nondrinker	0.064 (-0.065, 0.193)	0.338	0.954	-0.018 (-0.157, 0.121)	0.806	0.653	-0.048 (-0.205, 0.109)	0.552	0.671
	Drinker	0.017 (-0.330, 0.364)	0.925		0.049 (-0.323, 0.421)	0.798		0.116 (-0.364, 0.596)	0.636	
Neutrophils	Nondrinker	0.069 (-0.031, 0.169)	0.176	0.048	-0.008 (-0.116, 0.100)	0.889	0.234	-0.004 (-0.126, 0.118)	0.947	0.445
	Drinker	-0.044 (-0.152, 0.064)	0.420		-0.046 (-0.162, 0.070)	0.435		-0.040 (-0.191, 0.111)	0.604	
Monocytes	Nondrinker	0.016 (0.004, 0.028)	0.007	0.294	0.003 (-0.009, 0.015)	0.660	0.837	0.006 (-0.008, 0.020)	0.411	0.643
	Drinker	0.008 (-0.004, 0.020)	0.222		0.004 (-0.010, 0.018)	0.525		0.005 (-0.013, 0.023)	0.555	
Lymphocytes	Nondrinker	0.045 (-0.006, 0.096)	0.081	0.030	0.068 (0.013, 0.123)	0.014	0.033	0.020 (-0.043, 0.083)	0.522	0.328
	Drinker	0.025 (-0.030, 0.080)	0.364		0.026 (-0.033, 0.085)	0.378		0.079 (0.005, 0.153)	0.04	
RBC	Nondrinker	0.005 (-0.026, 0.036)	0.747	0.675	0.018 (-0.017, 0.053)	0.302	0.834	0.028 (-0.011, 0.067)	0.158	0.584
	Drinker	-0.011 (-0.038, 0.016)	0.434		0.005 (-0.024, 0.034)	0.731		-1.49×10^{-4} (-0.037, 0.037)	0.994	
Hemoglobin	Nondrinker	-0.140 (-1.046, 0.766)	0.761	0.738	-0.013 (-0.989, 0.963)	0.979	0.723	0.400 (-0.692, 1.492)	0.473	0.858
	Drinker	0.371 (-0.487, 1.229)	0.398		0.501 (-0.430, 1.432)	0.293		0.494 (-0.704, 1.692)	0.419	
Hematocrit	Nondrinker	0.072 (-0.210, 0.354)	0.617	0.502	0.100 (-0.204, 0.404)	0.521	0.278	0.255 (-0.090, 0.600)	0.148	0.550
	Drinker	0.137 (-0.096, 0.370)	0.250		0.222 (-0.029, 0.473)	0.084		0.201 (-0.124, 0.526)	0.226	
Platelet	Nondrinker	1.900 (-1.753, 5.553)	0.309	0.552	0.121 (-3.836, 4.078)	0.952	0.635	0.354 (-4.119, 4.827)	0.877	0.696
	Drinker	0.934 (-2.804, 4.672)	0.625		-0.345 (-4.375, 3.685)	0.867		1.075 (-4.127, 6.277)	0.686	
MPV	Nondrinker	-0.001 (-0.064, 0.062)	0.986	0.736	0.025 (-0.042, 0.092)	0.473	0.435	0.016 (-0.060, 0.092)	0.683	0.499
	Drinker	0.013 (-0.050, 0.076)	0.683		-0.002 (-0.071, 0.067)	0.955		0.057 (-0.031, 0.145)	0.206	

Note: Abbreviation: WBC, white blood cell; RBC, red blood cell; MPV, mean platelet volume.

[a] Generalized linear model with adjustment for age, sex, factory location, BMI, smoking status, pack-years of smoking, the corresponding baseline hematologic parameters, and other BTX components;

[b] $P_{interaction}$ were calculated by modeling an interaction term of single BTX components and drinking status in generalized linear models, with adjustment for age, sex, factory location, BMI, smoking status, pack-years of smoking, and the corresponding baseline hematologic parameters.

Table S11. Associations of BTX CE levels with decline in hematologic parameters stratified by BMI (kg/m^2).

Decline in hematologic parameters	BMI groups	Benzene			Toluene			Xylene		
		β (95% CI)	P_{trend}[a]	$P_{interaction}$[b]	β (95% CI)	P_{trend}[a]	$P_{interaction}$[b]	β (95% CI)	P_{trend}[a]	$P_{interaction}$[b]
WBC	<24	-0.005 (-0.285, 0.275)	0.974	0.403	-0.077 (-0.387, 0.233)	0.624	0.253	-0.071 (-0.418, 0.276)	0.690	0.436
	≥24	0.118 (-0.025, 0.261)	0.106		0.141 (-0.008, 0.290)	0.066		0.174 (-0.020, 0.368)	0.079	
Neutrophils	<24	-0.017 (-0.111, 0.077)	0.722	0.012	-0.101 (-0.205, 0.003)	0.057	0.001	-0.080 (-0.198, 0.038)	0.177	0.057
	≥24	0.065 (-0.049, 0.179)	0.263		0.070 (-0.050, 0.190)	0.248		0.083 (-0.072, 0.238)	0.291	
Monocytes	<24	0.010 (-0.002, 0.022)	0.103	0.323	0.002 (-0.012, 0.016)	0.742	0.772	0.005 (-0.009, 0.019)	0.508	0.370
	≥24	0.013 (0.001, 0.025)	0.030		0.005 (-0.007, 0.017)	0.474		0.005 (-0.011, 0.021)	0.587	
Lymphocytes	<24	0.020 (-0.027, 0.067)	0.400	0.291	0.016 (-0.037, 0.069)	0.554	0.029	0.010 (-0.049, 0.069)	0.739	0.023
	≥24	0.051 (-0.010, 0.112)	0.100		0.086 (0.023, 0.149)	0.008		0.093 (0.011, 0.175)	0.027	
RBC	<24	0.003 (-0.024, 0.030)	0.823	0.632	0.018 (-0.013, 0.049)	0.263	0.980	0.014 (-0.021, 0.049)	0.435	0.296
	≥24	-0.014 (-0.045, 0.017)	0.382		0.002 (-0.031, 0.035)	0.928		0.015 (-0.028, 0.058)	0.511	
Hemoglobin	<24	0.495 (-0.330, 1.320)	0.239	0.082	0.417 (-0.500, 1.334)	0.373	0.356	0.349 (-0.672, 1.370)	0.504	0.649
	≥24	-0.448 (-1.397, 0.501)	0.355		-0.113 (-1.109, 0.883)	0.824		0.537 (-0.753, 1.827)	0.415	
Hematocrit	<24	0.285 (0.038, 0.532)	0.024	0.342	0.257 (-0.021, 0.535)	0.070	0.812	0.234 (-0.078, 0.546)	0.142	0.271
	≥24	-0.042 (-0.316, 0.232)	0.766		0.121 (-0.163, 0.405)	0.406		0.314 (-0.054, 0.682)	0.097	
Platelet	<24	0.508 (-3.100, 4.116)	0.783	0.261	-2.718 (-6.760, 1.324)	0.188	0.052	-1.780 (-6.327, 2.767)	0.443	0.298
	≥24	2.438 (-1.268, 6.144)	0.198		2.848 (-1.001, 6.697)	0.148		4.499 (-0.491, 9.489)	0.078	
MPV	<24	0.024 (-0.037, 0.085)	0.444	0.375	0.036 (-0.033, 0.105)	0.299	0.411	0.073 (-0.003, 0.149)	0.062	0.507
	≥24	-0.026 (-0.089, 0.037)	0.421		-0.021 (-0.086, 0.044)	0.516		-0.036 (-0.120, 0.048)	0.403	

Note: Abbreviation: WBC, white blood cell; RBC, red blood cell; MPV, mean platelet volume.

[a] Generalized linear model with adjustment for age, sex, factory location, smoking status, pack-years of smoking, drinking status, and the corresponding baseline hematologic parameters;

[b] $P_{interaction}$ were calculated by modeling an interaction term of single BTX components and BMI groups in generalized linear models, with adjustment for age, sex, factory location, smoking status, pack-years of smoking, drinking status, and the corresponding baseline hematologic parameters.

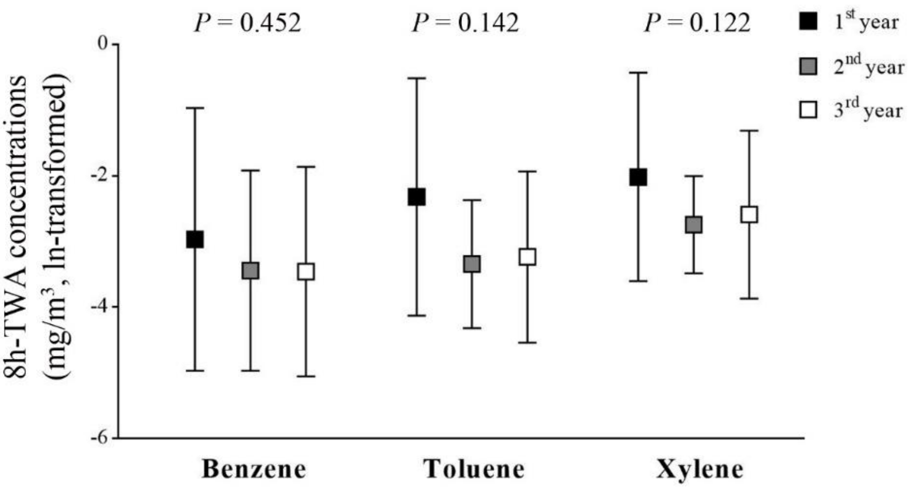

Figure S1. Comparisons of annual average BTX 8h-TWA concentrations using generalized estimating equation models.

Note: Abbreviation: 8h-TWA, 8-hour time-weighted average concentrations.

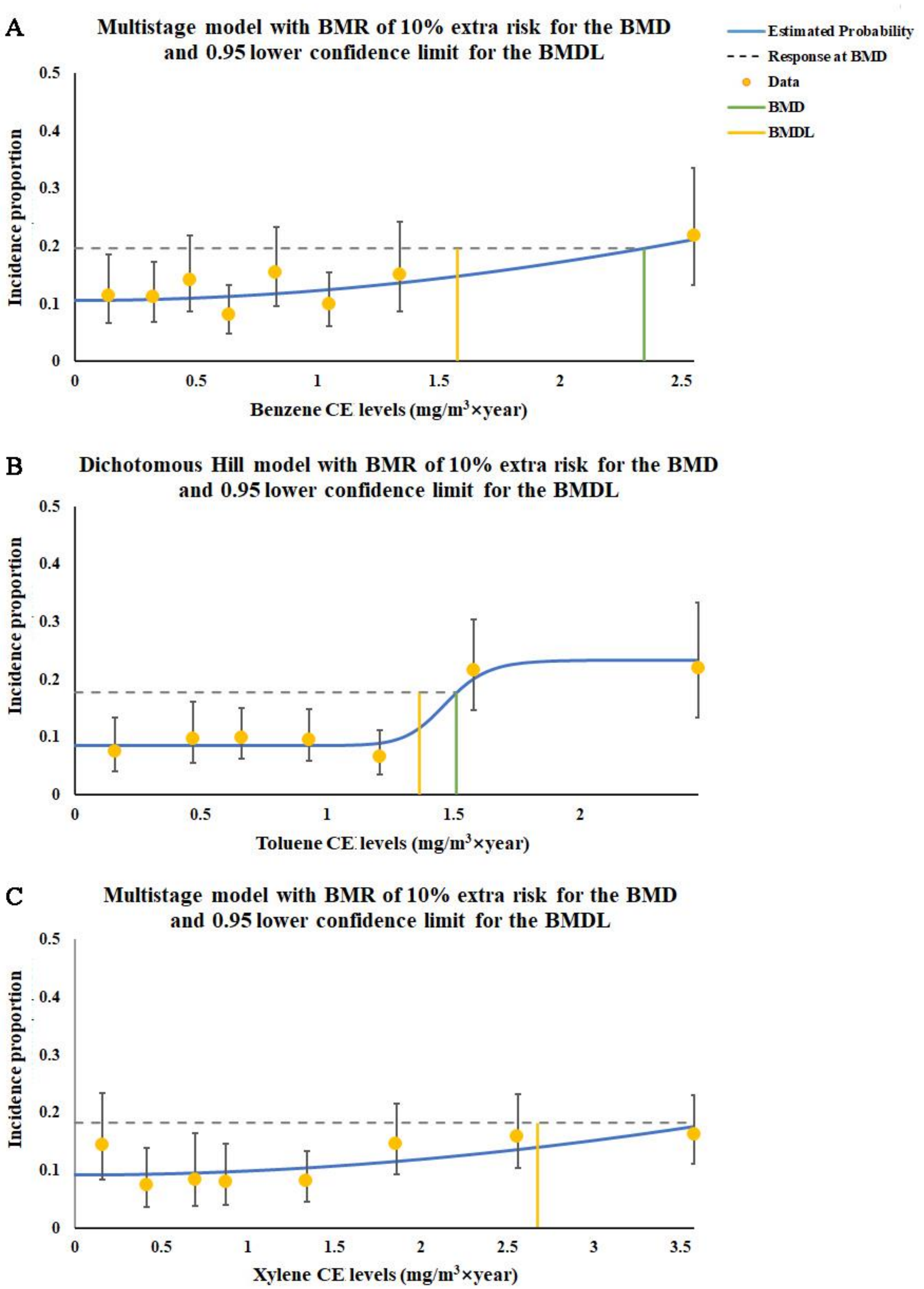

Figure S2. Dose-response curves and estimated BMD and BMDL of CE levels of benzene (A), toluene (B), and xylene (C) for the incidence proportions of hematological damage.

Note: Abbreviation: BMR, benchmark response; BMD, benchmark doses;

BMDL, lower confidence limit of BMD; CE, cumulative exposure.

The X axis described the BTX CE levels (mg/m^3 × year), and the Y axis described the incidence proportions of hematological damages.
